# Supplementary material for: IoT-based demand-side energy management: Enhancing peak hour efficiency through automated control of appliances
Source: PLoS One. 2026 May 4;21(5):e0347990. doi: 10.1371/journal.pone.0347990 (PMC13138677; doi:10.1371/journal.pone.0347990)
Supplement: S1 File — (DOCX) [file pone.0347990.s001.docx]

Supporting Information files

S1 Fig communication interfaces and control.


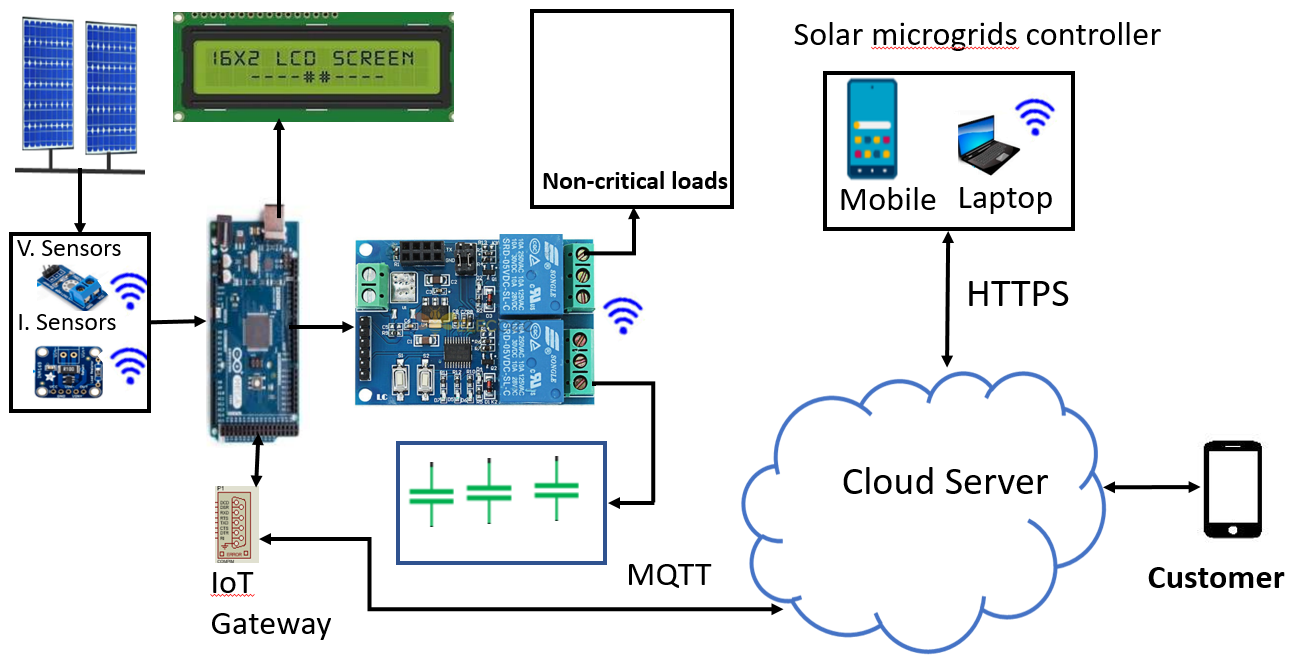


S2 Table for Fig 9

| Time (HRS) | Energy (kWh) |
| --- | --- |
| 1 | 52 |
| 2 | 55 |
| 3 | 58 |
| 4 | 65 |
| 5 | 71 |
| 6 | 83 |
| 7 | 80 |
| 8 | 110 |
| 9 | 125 |
| 10 | 142 |
| 11 | 150 |
| 12 | 138 |
| 13 | 120 |
| 14 | 114 |
| 15 | 101 |
| 16 | 92 |
| 17 | 62 |
| 18 | 73 |
| 19 | 79 |
| 20 | 57 |
| 21 | 49 |
| 22 | 47 |
| 23 | 50 |
| 24 | 45 |

| Days | Energy (kWh) |
| --- | --- |
| 1 | 644 |
| 2 | 691 |
| 3 | 812 |
| 4 | 756 |
| 5 | 1013 |
| 6 | 669 |
| 7 | 549 |
| 8 | 506 |
| 9 | 560 |
| 10 | 459 |
| 11 | 633 |
| 12 | 768 |
| 13 | 363 |
| 14 | 513 |
| 15 | 443 |
| 16 | 614 |
| 17 | 485 |
| 18 | 428 |
| 19 | 503 |
| 20 | 694 |
| 21 | 537 |
| 22 | 369 |
| 23 | 475 |
| 24 | 379 |
| 25 | 638 |
| 26 | 689 |
| 27 | 543 |
| 28 | 578 |
| 29 | 609 |
| 30 | 716 |
| 31 | 697 |

S3 Table for Fig 10

| S4 Table for Fig 11   \| Days \| Daily energy consumption at Brewery sections for March 2023 in kWh \| \| \| \| \| \| \| \| \| --- \| --- \| --- \| --- \| --- \| --- \| --- \| --- \| --- \| \| MALT TOWER \| BREW. H P \| BEER PC \| YEAST FC \| Fv&SV \| BREW HA \| MALT T SL \| MCC BR \| \| 1 \| 85.3 \| 90.6 \| 87.5 \| 81.5 \| 67.3 \| 83.4 \| 78.6 \| 69.8 \| \| 2 \| 91.3 \| 96.6 \| 93.8 \| 86.1 \| 73.4 \| 89.5 \| 84.9 \| 75.4 \| \| 3 \| 106.2 \| 112.9 \| 108.5 \| 103.4 \| 88.1 \| 105.8 \| 98.6 \| 88.5 \| \| 4 \| 73.6 \| 79.1 \| 75.5 \| 69.7 \| 55.4 \| 71.8 \| 66.3 \| 57.6 \| \| 5 \| 68.4 \| 73.5 \| 70.1 \| 62.3 \| 50.7 \| 66.5 \| 61.6 \| 52.9 \| \| 6 \| 88.9 \| 83.6 \| 91.3 \| 84.5 \| 80.8 \| 86.2 \| 81.1 \| 72.6 \| \| 7 \| 103.6 \| 112.7 \| 107.1 \| 99.5 \| 85.3 \| 103.8 \| 86.4 \| 87.6 \| \| 8 \| 131.8 \| 136.3 \| 134.5 \| 127.7 \| 113.6 \| 129.4 \| 125.2 \| 114.5 \| \| 9 \| 83.2 \| 94.5 \| 68.1 \| 86.7 \| 76.3 \| 84.6 \| 74.4 \| 65.2 \| \| 10 \| 101.7 \| 106.3 \| 102.8 \| 94.7 \| 93.6 \| 98.3 \| 90.2 \| 80.4 \| \| 11 \| 49.4 \| 55.2 \| 53.1 \| 43.6 \| 41.2 \| 47.5 \| 39.7 \| 33.3 \| \| 12 \| 62.8 \| 67.3 \| 63.7 \| 54.2 \| 53.1 \| 59.4 \| 50.6 \| 47.9 \| \| 13 \| 75.1 \| 80.3 \| 77.6 \| 68.7 \| 66.8 \| 73.9 \| 64.4 \| 53.2 \| \| 14 \| 68.1 \| 78.8 \| 73.5 \| 61.4 \| 60.2 \| 67.1 \| 57.6 \| 46.3 \| \| 15 \| 60.5 \| 69.4 \| 62.6 \| 52.8 \| 50.5 \| 56.9 \| 49.1 \| 41.2 \| \| 16 \| 85.7 \| 88.6 \| 82.7 \| 75.2 \| 71.5 \| 78.3 \| 69.1 \| 62.9 \| \| 17 \| 64.6 \| 74.4 \| 67.5 \| 58.1 \| 55.6 \| 63.3 \| 53.7 \| 47.8 \| \| 18 \| 58.5 \| 67.2 \| 60.3 \| 51.6 \| 48.7 \| 55.4 \| 46.2 \| 40.1 \| \| 19 \| 67.2 \| 80.4 \| 70.5 \| 61.7 \| 47.2 \| 65.1 \| 56.3 \| 54.6 \| \| 20 \| 90.7 \| 99.5 \| 94.7 \| 85.2 \| 76.1 \| 74.6 \| 90.8 \| 82.4 \| \| 21 \| 72.1 \| 87.4 \| 74.5 \| 64.3 \| 55.8 \| 54.1 \| 70.2 \| 58.6 \| \| 22 \| 51.4 \| 59.6 \| 53.8 \| 43.2 \| 35.7 \| 33.5 \| 50.1 \| 41.7 \| \| 23 \| 64.7 \| 73.3 \| 67.5 \| 57.8 \| 48.6 \| 46.2 \| 63.1 \| 53.8 \| \| 24 \| 91.8 \| 100.2 \| 97.7 \| 83.1 \| 74.3 \| 72.8 \| 88.5 \| 80.6 \| \| 25 \| 85.3 \| 97.1 \| 89.9 \| 75.4 \| 67.2 \| 66.6 \| 82.7 \| 73.8 \| \| 26 \| 52.5 \| 50.4 \| 53.7 \| 63.5 \| 38.4 \| 34.1 \| 43.6 \| 42.8 \| \| 27 \| 73.9 \| 88.1 \| 75.6 \| 54.4 \| 53.1 \| 71.2 \| 61.6 \| 65.1 \| \| 28 \| 76.5 \| 91.8 \| 83.6 \| 70.1 \| 63.3 \| 57.4 \| 69.7 \| 65.6 \| \| 29 \| 81.1 \| 94.8 \| 83.5 \| 73.4 \| 64.7 \| 62.6 \| 78.7 \| 70.2 \| \| 30 \| 95.4 \| 91.7 \| 97.8 \| 87.3 \| 78.5 \| 86.1 \| 94.4 \| 84.8 \| \| 31 \| 92.3 \| 97.2 \| 94.5 \| 84.1 \| 70.6 \| 86.2 \| 91.7 \| 80.4 \| |  |  |  |  |  |  |  |  |
| --- | --- | --- | --- | --- | --- | --- | --- | --- | --- | --- | --- | --- | --- | --- | --- | --- | --- | --- | --- | --- | --- | --- | --- | --- | --- | --- | --- | --- | --- | --- | --- | --- | --- | --- | --- | --- | --- | --- | --- | --- | --- | --- | --- | --- | --- | --- | --- | --- | --- | --- | --- | --- | --- | --- | --- | --- | --- | --- | --- | --- | --- | --- | --- | --- | --- | --- | --- | --- | --- | --- | --- | --- | --- | --- | --- | --- | --- | --- | --- | --- | --- | --- | --- | --- | --- | --- | --- | --- | --- | --- | --- | --- | --- | --- | --- | --- | --- | --- | --- | --- | --- | --- | --- | --- | --- | --- | --- | --- | --- | --- | --- | --- | --- | --- | --- | --- | --- | --- | --- | --- | --- | --- | --- | --- | --- | --- | --- | --- | --- | --- | --- | --- | --- | --- | --- | --- | --- | --- | --- | --- | --- | --- | --- | --- | --- | --- | --- | --- | --- | --- | --- | --- | --- | --- | --- | --- | --- | --- | --- | --- | --- | --- | --- | --- | --- | --- | --- | --- | --- | --- | --- | --- | --- | --- | --- | --- | --- | --- | --- | --- | --- | --- | --- | --- | --- | --- | --- | --- | --- | --- | --- | --- | --- | --- | --- | --- | --- | --- | --- | --- | --- | --- | --- | --- | --- | --- | --- | --- | --- | --- | --- | --- | --- | --- | --- | --- | --- | --- | --- | --- | --- | --- | --- | --- | --- | --- | --- | --- | --- | --- | --- | --- | --- | --- | --- | --- | --- | --- | --- | --- | --- | --- | --- | --- | --- | --- | --- | --- | --- | --- | --- | --- | --- | --- | --- | --- | --- | --- | --- | --- | --- | --- | --- | --- | --- | --- | --- | --- | --- | --- | --- | --- | --- | --- | --- | --- | --- | --- | --- | --- | --- | --- | --- | --- | --- | --- | --- | --- | --- | --- | --- | --- | --- | --- | --- | --- | --- | --- | --- | --- | --- | --- | --- | --- |
|  |  |  |  |  |  |  |  |  |
|  |  |  |  |  |  |  |  |  |
|  |  |  |  |  |  |  |  |  |
|  |  |  |  |  |  |  |  |  |
| S5 Table for Fig 12   \| Brewery Sub-Sections \| Energy consumption in kWh for days of a second week of March 2023 \| \| \| \| \| \| \| \| --- \| --- \| --- \| --- \| --- \| --- \| --- \| --- \| \| Monday \| Tuesday \| Wednesday \| Thursday \| Friday \| Saturday \| Sunday \| \| Malt Tower \| 88.9 \| 103.6 \| 131.8 \| 83.2 \| 101.7 \| 58.8 \| 49.4 \| \| Brew house plant \| 83.6 \| 112.7 \| 136.3 \| 94.5 \| 106.3 \| 60.3 \| 50.2 \| \| Beer process cellars \| 84.5 \| 99.5 \| 127.7 \| 86.7 \| 94.7 \| 54.2 \| 43.6 \| \| Yeast, Filter cellars \| 83.8 \| 98.9 \| 128.3 \| 87.4 \| 95.1 \| 53.8 \| 43.7 \| \| FV & SV \| 80.8 \| 85.3 \| 113.6 \| 76.3 \| 93.6 \| 53.1 \| 41.2 \| \| Brew house AUX \| 86.2 \| 103.8 \| 129.4 \| 84.6 \| 98.3 \| 57.4 \| 47.5 \| \| Malt Tower, S/light \| 81.1 \| 86.4 \| 125.2 \| 74.4 \| 90.2 \| 50.6 \| 39.7 \| \| MCC Brewhouse office \| 72.6 \| 87.6 \| 114.5 \| 65.2 \| 80.4 \| 47.9 \| 33.3 \| |  |  |  |  |  |  |  |  |
| S6 Table for Fig13 |  |  |  |  |  |  |  |  |
| \| Time (HRS) \| Time-Scheduled (kWh) \| Power-Scheduled (kWh) \| Critical Loads (kWh) \| \| --- \| --- \| --- \| --- \| \| 1 \| 60 \| 55 \| 80 \| \| 2 \| 60 \| 55 \| 80 \| \| 3 \| 60 \| 55 \| 80 \| \| 4 \| 50 \| 70 \| 56 \| \| 5 \| 63 \| 70 \| 56 \| \| 6 \| 62 \| 70 \| 56 \| \| 7 \| 50 \| 50 \| 55 \| \| 8 \| 50 \| 50 \| 55 \| \| 9 \| 130 \| 54 \| 55 \| \| 10 \| 130 \| 150 \| 120 \| \| 11 \| 130 \| 150 \| 120 \| \| 12 \| 130 \| 150 \| 120 \| \| 13 \| 130 \| 90 \| 80 \| \| 14 \| 70 \| 90 \| 80 \| \| 15 \| 70 \| 90 \| 80 \| \| 16 \| 55 \| 90 \| 80 \| \| 17 \| 55 \| 90 \| 65 \| \| 18 \| 55 \| 55 \| 65 \| \| 19 \| 72 \| 55 \| 76 \| \| 20 \| 72 \| 55 \| 76 \| \| 21 \| 72 \| 59 \| 76 \| \| 22 \| 90 \| 59 \| 65 \| \| 23 \| 90 \| 59 \| 65 \| \| 24 \| 90 \| 59 \| 65 \|   S7 Table for Fig 14   \| Time (HRS) \| Time-Scheduled (kWh) \| Power-Scheduled (kWh) \| Critical Loads (kWh) \| \| --- \| --- \| --- \| --- \| \| 1 \| 130 \| 55 \| 80 \| \| 2 \| 130 \| 55 \| 80 \| \| 3 \| 130 \| 55 \| 80 \| \| 4 \| 130 \| 70 \| 56 \| \| 5 \| 130 \| 70 \| 56 \| \| 6 \| 70 \| 90 \| 56 \| \| 7 \| 70 \| 90 \| 55 \| \| 8 \| 50 \| 90 \| 55 \| \| 9 \| 60 \| 54 \| 55 \| \| 10 \| 60 \| 55 \| 120 \| \| 11 \| 60 \| 55 \| 120 \| \| 12 \| 50 \| 55 \| 120 \| \| 13 \| 63 \| 59 \| 80 \| \| 14 \| 62 \| 59 \| 80 \| \| 15 \| 50 \| 59 \| 80 \| \| 16 \| 55 \| 50 \| 80 \| \| 17 \| 55 \| 150 \| 65 \| \| 18 \| 55 \| 150 \| 65 \| \| 19 \| 72 \| 150 \| 76 \| \| 20 \| 72 \| 90 \| 76 \| \| 21 \| 72 \| 70 \| 76 \| \| 22 \| 90 \| 70 \| 65 \| \| 23 \| 90 \| 70 \| 65 \| \| 24 \| 90 \| 70 \| 65 \| |  |  |  |  |  |  |  |  |
|  |  |  |  |  |  |  |  |  |
|  |  |  |  |  |  |  |  |  |
|  |  |  |  |  |  |  |  |  |
|  |  |  |  |  |  |  |  |  |
|  |  |  |  |  |  |  |  |  |
|  |  |  |  |  |  |  |  |  |
